# Complete genome sequencing of *Bacillus* sp. TK-2, analysis of its cold evolution adaptability

**DOI:** 10.1038/s41598-021-84286-7

**Published:** 2021-03-01

**Authors:** Lijun Shen, Xueli Zang, Ke Sun, Huan Chen, Xinying Che, Yang Sun, Gang Wang, Sitong Zhang, Guang Chen

**Affiliations:** 1 College of Life Sciences, Jilin Agricultural University, Changchun, China; 2 Key Laboratory of Straw Biology and Utilization, The Ministry of Education, Changchun, China; 3 School of Medicine and Food, Changchun Medical College, Changchun, China; 4 COFCO Biochemical Energy (Gongzhuling) Co., Ltd, Changchun, China

**Keywords:** Biotechnology, Evolution, Microbiology, Molecular biology, Environmental sciences, Energy science and technology

## Abstract

To date, a large number of *Bacillus* species from different sources have been identified. However, there are few investigations on genome information and evolutionary insights of *Bacillus* species from cold environments. *Bacillus* sp. TK-2, isolated from the soil of Changbai Mountain, is a gram-positive bacterium with cold adaptation characteristics. In this study, we present the annotated complete genome sequence of *Bacillus* sp. TK-2. The genome comprised 5,286,177 bp with a GC content of 35.88%, 5293 protein-encoding genes, 32 rRNA, and 77 tRNA. Numerous genes related to cold adaptation were detected in the genome of *Bacillus* sp. TK-2, mainly involving in energy supply, regulation of cell membrane fluidity, antioxidant, and molecular chaperones. In addition, the strain TK-2 classified in the *Bacillus* groups was distributed on a terminal branch with *Bacillus cereus* A1 by Blastn and phylogenetic analysis in NCBI database. Complete genome sequences of the strain TK-2 and *Bacillus cereus* A1 were compared by the online tool “Average Nucleotide Identity”, showing that the average nucleotide identity of these two strains was 98.26%. In parallel, A comparative analysis of the genomes of both *Bacillus* sp. TK-2 and *Bacillus cereus* A1 was conducted. Through the analysis of core and specific genes with cd-hit, it was found that the two strains had 5691 pan gene, 4524 core gene, and 1167 specific gene clusters. Among the 624 specific gene clusters of *Bacillus* sp. TK-2, some cold tolerance genes were detected, which implied the unique adaptability of *Bacillus* sp. TK-2 in long-term low temperature environments. Importantly, enzyme-encoding genes related to the degradation of polysaccharides such as cellulose and hemicellulose were detected in the 477 CAZyme genes of this genome. This work on sequencing and bioinformatics analysis of the complete sequence of *Bacillus* sp. TK-2 promote the application and in-depth research of low-temperature biotechnology.

## Introduction

In recent years, low-temperature microorganisms have become a focus of the field of extreme environmental microbiology, especially their physiological metabolism and cold adaptation mechanisms, which are of great significance for promoting the establishment and application of low-temperature biotechnology. Low-temperature microorganisms are widely distributed, mainly found in deep seas, lakes, glaciers, and perennial cold soils^1^. They will adopt a series of strategies to maintain their normal growth and metabolism in a cold environment. For example, the increase in the proportion of unsaturated fatty acids can regulate the fluidity of cell membranes. The synthesis of compatible solutes (glycine, betaine, glycerin, trehalose, sucrose, mannitol, sorbitol) has the function of antifreeze protection and acts as a carbon and nitrogen source for microorganisms. The mass production of cold stress proteins plays a positive role in the process of cold adaptation of microorganisms^2,3^. Currently, more and more studies have applied multi-omics technology to explore the mechanism of bacteria adapting to the cold environment^4,5^.

Complete genome sequencing technology has a good application prospect in the discovery of genome sequence information of unknown bacteria and the exploration of critical functional genes^6^. Tolerance potential to drought and salt stress of *Bacillus halotolerans* was revealed by complete genome sequencing^7^. Through complete genome sequencing of *Bacillus thuringiensis*, key genes with insect resistance functions were found, providing valuable genetic information for subsequent industrial applications^8^. Some of the *Bacillus* sp. have diarrheal toxin genes, which cause adverse effects on food and environment. Complete genome sequencing can be used to provide a clear genetic background for distinguishing toxin-producing strains^9^. *Bacillus* strains derived from rice wine and grape leaves that inhibited the growth of plant pathogens were discovered. The key gene clusters that controlled plant pathogens were predicted through their complete genome sequencing, which provided evidence for revealing their specific disease resistance mechanisms^10,11^. Related genes involved in the hydrolysis of lignocellulose, starch, and other polysaccharides were detected by genomic sequencing of *Bacillus* sp, implying the application potential of this strain in the degradation of polysaccharides in lignocellulose^12^.

Due to a large number of studies on microbial genome sequencing, it is essential to obtain more genome information of low-temperature microorganisms for the development of cold biotechnology. In this study, the strain TK-2, derived from Changbai Mountain's perennially low-temperature soil, was initially classified as *Bacillus* sp. by 16S rDNA sequence analysis. Through the analysis of growth characteristics, *Bacillus* sp. TK-2 with cellulase and xylanase activity has the best growth state at 15 °C, which is beneficial to increase the degradation rate of biomass in cold environments. Based on this, the complete genome sequencing and comparative analysis of *Bacillus* sp. TK-2 were conducted, which provided evidence for potential genetic basis for its adaptation to a cold environment.

## Results and discussion

**Figure 1:**
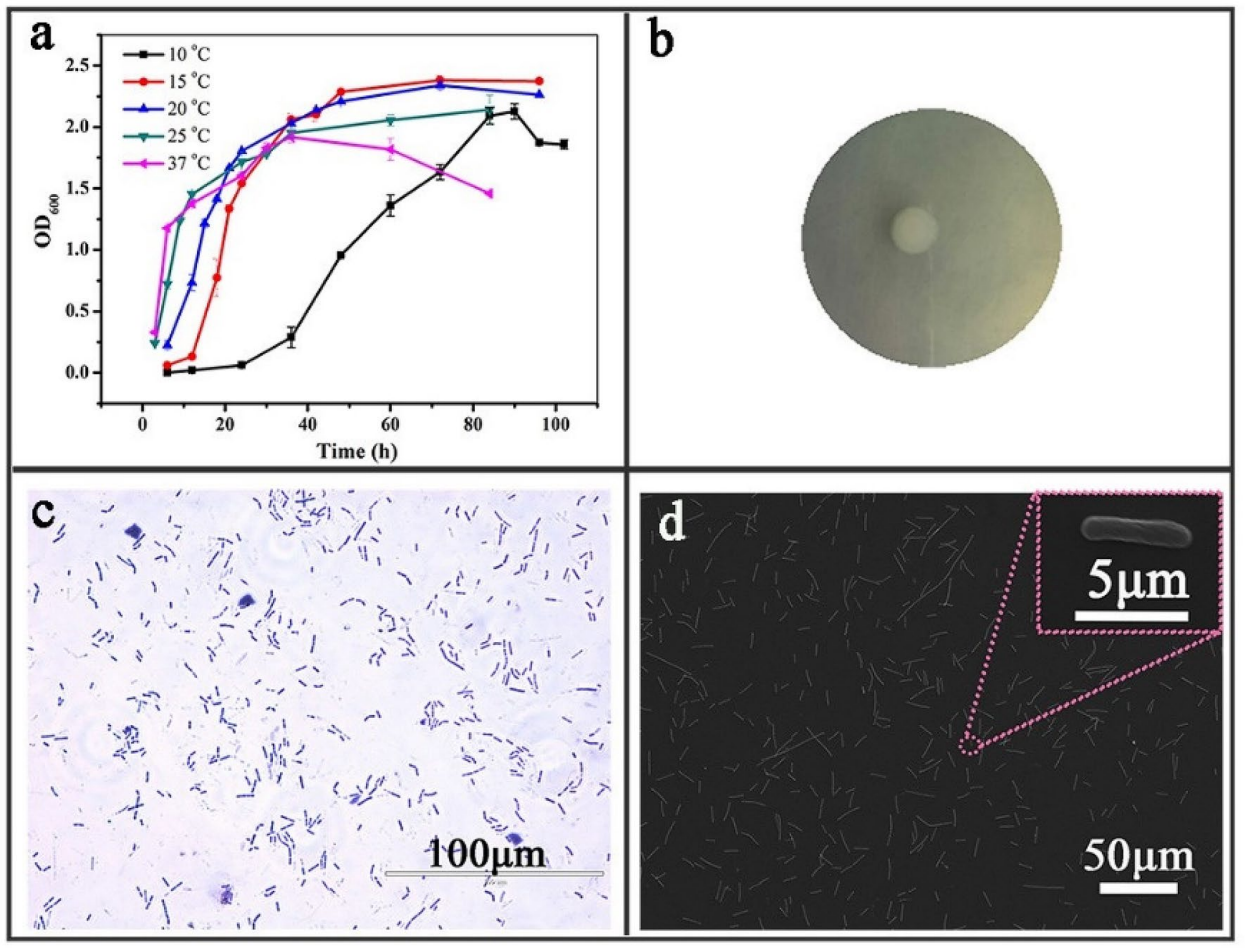
(**a**) The growth curve of the strain TK-2 at different temperatures. (**b**) The morphology of TK-2 on the medium. (**c**,**d**) The optical photos of the strain TK-2 under microscope and SEM.

The growth of the strainTK-2 was measured at different temperatures of 10, 15, 20, 25, 30, and 37 °C. It can be seen that the optimal growth temperature of *Bacillus* sp.TK-2 was 15 °C (Fig. 1a). The strain TK-2 was in the initial growth period from 0 to 12 h, entered the logarithmic growth period after 12 h, and reached the stable period at 48 h. The growth rate of the strain TK-2 at 10 °C was significantly lower than that at 15 °C. It was in the initial growth phase from 0 to 48 h, entered the logarithmic phase of growth at 48 h, and began to stabilize at 84 h. The whole growth cycle was longer. The growth state at 20 °C of the strain TK-2 was slightly worse than that at 15 °C. In addition, when it exceeded 20 °C (25 °C and 37 °C), the strain quickly entered the logarithmic growth phase at 3 h, and began the stable phases at 18 h. The value of OD_600_ was significantly lower than that of other temperatures. In general, it could be seen showed that 15 °C was the optimal growth temperature for the strain TK-2. Whether it was higher or lower than this temperature, the growth of strain TK-2 was inhibited to varying extents. This is consistent with the results described by previous study^13^. These results provided valuable insights into the growth characteristics of cold-tolerant bacteria at low temperature.

The strainTK-2 was a coryneform bacterium with large volume, opaque, rough surface, and white frosted glass (Fig. 1b,c,d). Through analysis of physiological and biochemical characteristics, it was found that the strainTK-2 was a gram-positive bacterium, which could hydrolyze starch, liquefy gelatin, and produced fluorescent pigments, but did not produce acid from mannitol. More physiological and biochemical characteristics are shown in Table 1. The “+” represents positive, and “−” represents negative in Table 1. The 16S rDNA method was used for molecular identification. The 16S rDNA sequence of the strain TK-2 was amplified by PCR using primer 27F/1492R and a template (the genomic DNA extracted from *Bacillus* sp. TK-2) (Fig. S1). The purified PCR products that were examined by agarose gel electrophoresis were sequenced to obtain the sequence (three parallels). The nucleotide sequence of 16S rDNA (Fig. S2) was presented in GenBank database under the accession number MW435590. Compared with all available 16S rDNA sequences in the NCBI database by using blastn, the highest similarity result was *Bacillus* sp. Combined with morphological and physiological and biochemical analysis, the strain TK-2 was initially determined to belong to the genus *Bacillus*. In addition, genome sequencing was performed to understand the genetic evolution information of *Bacillus* sp. TK-2 at low temperature.

**Table 1:** Determination of physiological and biochemical characteristics of the strain TK-2.

Tests	*Bacillus* sp. TK-2	Tests	*Bacillus* sp. TK-2
Gram staining	+	Indole production	+
Fluorescent pigment	+	Gelatin liquefaction	+
Methyl red	+	Hippuric acid	+
Contact angle	+	V-P determination	+
Pyocyanin	+	Nitrate reduction	+
Starch hydrolysis	+	Mannitol acid production	−

**Figure 2:**
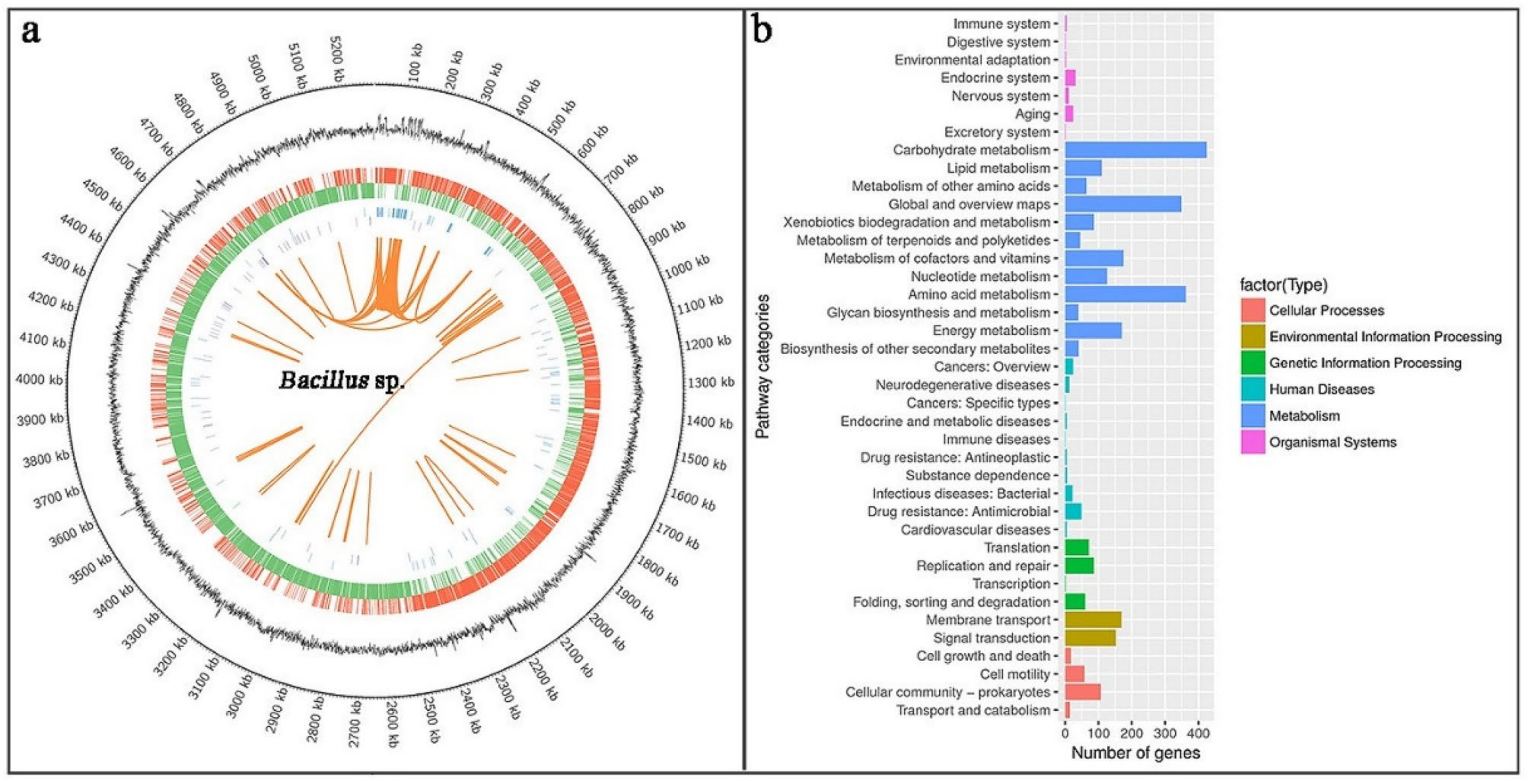
(**a**) Genomic circle diagram of *Bacillus* sp. TK-2. The genome circle diagram has seven circles, from the outer circle to the inner circle, and the information displayed in each circle is the genome position, GC content, and coding gene on the positive strand. (Red), the coding gene on the negative strand (green mark), the ncRNA information on the positive strand (blue), and the ncRNA information on the negative strand (purple), which are the long-segment repetitive sequence information in the genome (orange). (**b**) Details of KEGG of *Bacillus* sp. TK-2. There are six categories, as shown on the right side of Fig. 2b, each category is divided into secondary classification system, X-axis is the number of genes, Y-axis is biological pathway Secondary classification, different colors are used to distinguish the primary classification of biological pathways.

**Figure 3:**
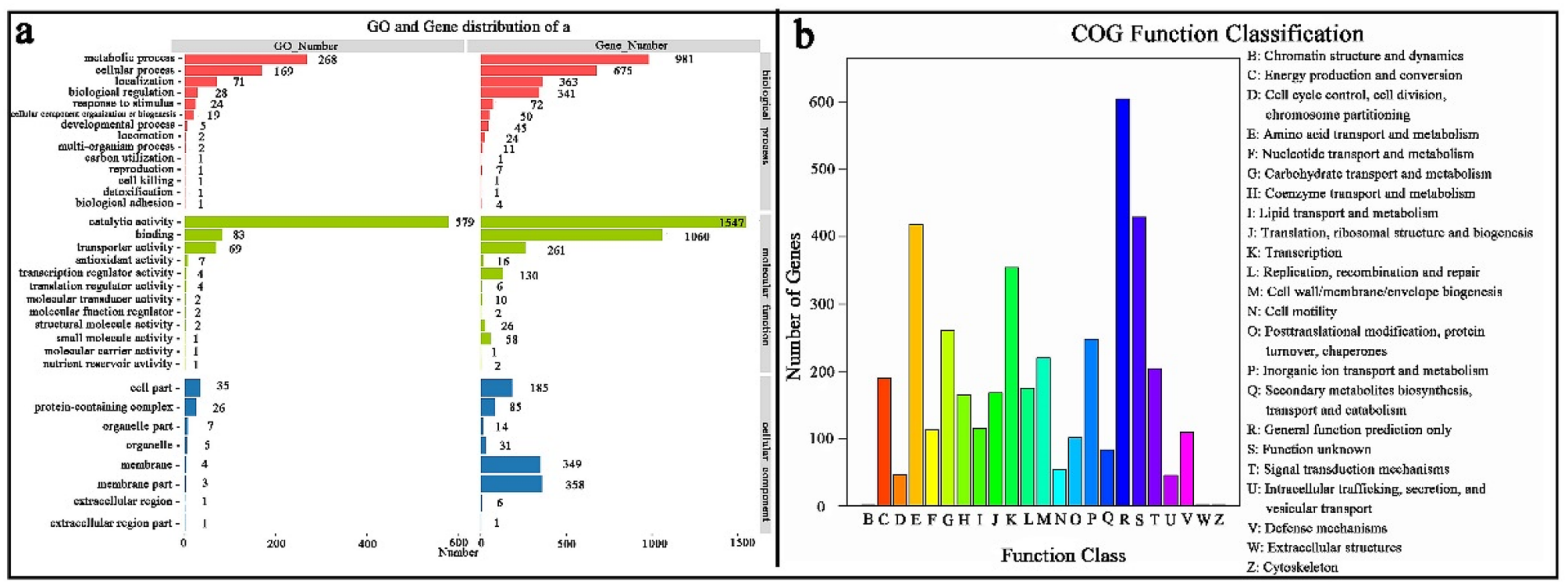
(**a**) GO function classification of *Bacillus* sp. TK-2. The X-axis is the number of genes, Y-axis is GO term, and different colors are used to distinguish biological processes, cell components, and molecular functions. (**b**) COG functional classification of genes, the X-axis is the functional classification of COG, the Y-axis is the number of genes annotated in each classification, and the explanation of each classification is marked on the right side of (**b**).

In recent years, due to the application potential of extreme microorganisms and their products in modern bioengineering, a large number of studies on the sequencing of microbial genomes with excellent performance from extreme environments have emerged^8,12^. Therefore, complete-genome sequencing of *Bacillus* sp. TK-2 was carried out to provide evidence for the genetic basis of how the strain TK-2 responded in cold environments. The results showed that the genome comprised 5,286,177 bp with the average length of coding genes of 838.83 bp, a GC content of 35.88%, 5293 protein-encoding genes, 32 rRNAs, and 77 tRNAs (Table 2; Fig. 2). All genes are functionally annotated in Nr (Non-Redundant Protein), GO (Gene Ontology), COG (Clusters of Orthologous Groups), and KEGG (Kyoto Encyclopedia of Genes and Genomes) databases. Biological metabolic pathways are divided into 6 categories by the KEGG database. In the strain TK-2, there are 2935 genes annotated to function and metabolic pathways, accounting for 55.5% of the total number of encoded genes, of which have 67.8% metabolic, 10.9% environmental information genes, 6.7% cellular processes, 7.5% genetic information processing, 4.4% human diseases, and 2.7% organismal systems. Each of these six categories has its sub-category, so it can be discovered which genes play a common role in the target metabolite, which is up- or down-regulated (Fig. 2). The GO database divides genomic information into three parts: molecular function (M), biological process (P); cellular component (C)^14,15^. In *Bacillus* sp. TK-2, 2724 genes were annotated to the GO database, accounting for 51.5% of the entire encoded genes. It can be seen that the gene products of this strain are mainly in molecular functions. The statistical results were shown in Fig. 3a. The COG database was used to classify the phylogenetic relationship of proteins encoded by the complete genome of the strain. There were 4106 annotated genes, accounting for 77.6% of the total number of genes, of which 10.2% amino acid transport and metabolism (E) (418), 6.35% carbohydrate transport and metabolism (G) (261), 8.6% transcription (K) (354). Also, it includes 14.7% genes with general function prediction and 10.4% genes with unknown function, which is worthy of further functional research (Fig. 3b). Based on the good growth adaptability of the strain TK-2 at low temperature, combined with the functional information that has been annotated in these databases, the proteins and metabolic pathways related to cold tolerance of the strainTK-2 are summarized and discussed.

**Table 2:** Basic features of *Bacillus* sp. TK-2 genome.

Features	Value
Genome size (bp)	5,286,177
Average length of coding genes (bp)	838.83
(G + C) %	35.88%
The number of protein coding genes	5293
The number of rRNA genes	32
The number of tRNA genes	77
The number of other non-coding RNAs	123

The genes annotated in these databases of the strain TK-2 contained many proteins involved in cold adaptation. First, Energy was stored by the accumulation of glycogen^16^, gluconeogenesis^17^, and compatibility solute synthesis^18,19^ to improve the antifreeze ability of strains. Second, the up-regulation of fatty acid desaturase^20^, fatty acid metabolism regulator (FadR) and 3-hydroxy acyl-ACP dehydratase (FabA)^21^, mitogen-activated protein kinase (MAPK) signaling pathway^22^ plays an important role in maintaining the normal fluidity of cell membranes at low temperatures. Third, the accumulation of polyhydroxyalkanoate (PHA)^23^ and the regulation of some oxidases such as catalase, superoxide dismutase, and glutathione oxidase^24,25^ can cope with oxidative stress caused by low temperature. Fourth, the expression of molecular chaperones has been confirmed to be up-regulated at low temperatures, such as Hsp, Csp^26^, proteins involved in pH homeostasis (ATP synthase subunits AtpA and AtpB), stress response proteins (chaperone DnaK and GroEL)^27^. Besides, their expression is upregulated at low temperatures, such as proteins involved in amino acid transport and metabolism, carbohydrate transport and metabolism^28,29^. Some regulatory factors, and genes related to DNA replication, recombination, and modification^30^, which play a key protective role in the cold adaptation of strains. Extracellular polysaccharides are the composition of the extracellular polymer around bacterial cells, playing a considerable cellular function in dealing with cold and freezing^31^.

**Figure 4:**
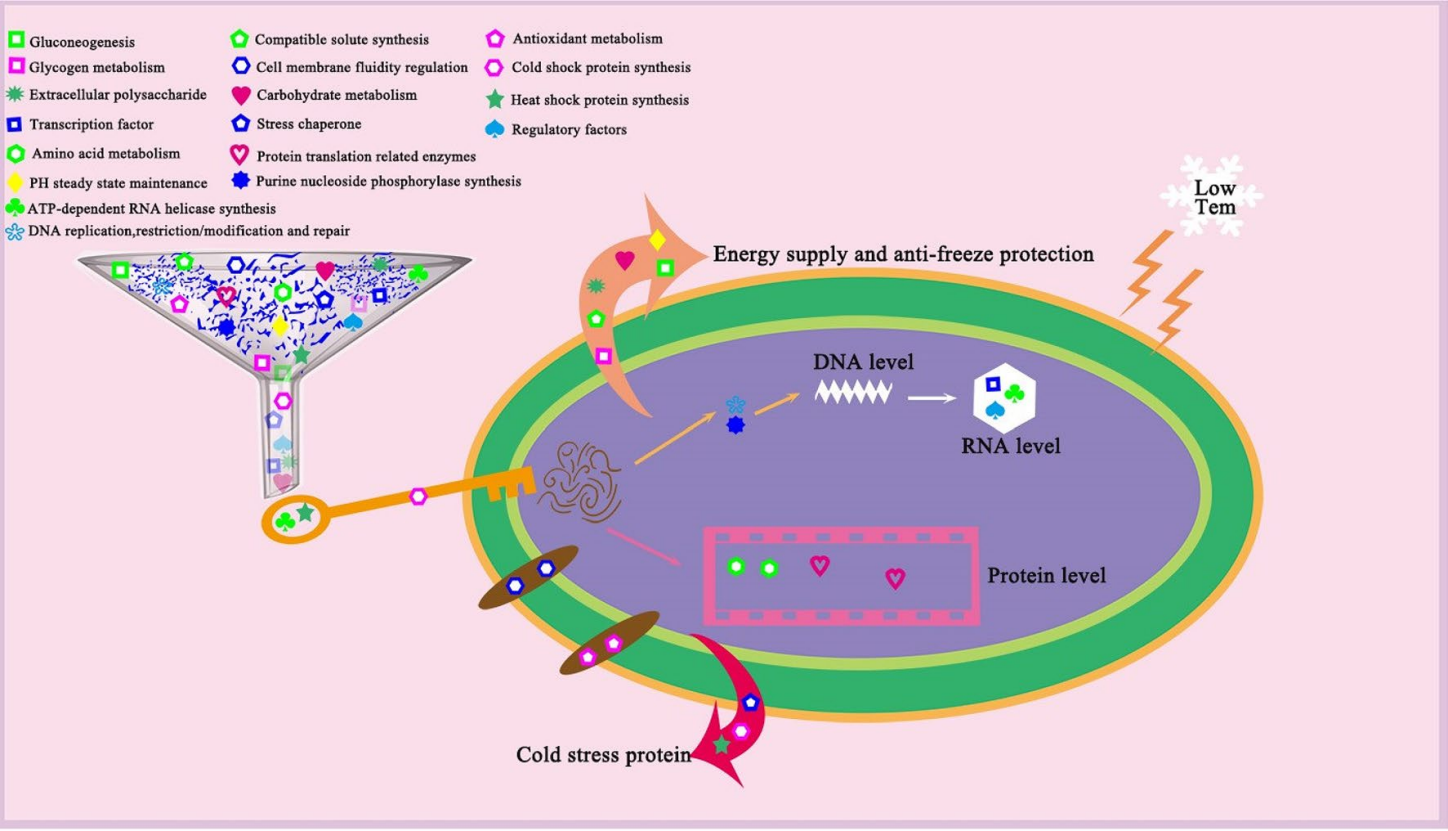
Strategies diagram for predicting cold tolerance of *Bacillus* sp. TK-2.

Some preliminary strategies for the strain were predicted to maintain normal growth and metabolism at low temperatures by a summary of the enzyme proteins and genes related to the cold adaptability of *Bacillus* sp. TK-2. As shown in Fig. 4, it mainly involves energy supply, regulation of cell membrane fluidity, antioxidant metabolism, and high expression of proteins at low temperatures, which promotes the maintenance of normal DNA, RNA, and protein interactions. Hence, it is speculated that this is the key to adapting to the low temperature of *Bacillus* sp. TK-2^32^. Genomic analysis not only predicts the low-temperature adaptability strategy of the strain, but also provides relevant genetic information for subsequent functional verification and discovery of new genes. Also, whether some genes (unknown function) that are not classified in KEGG, COG, GO and other databases are involved in low-temperature adaptability of *Bacillus* sp. TK-2 will be revealed and discussed in future studies through a combination of multiple omics analyses.

**Figure. 5:**
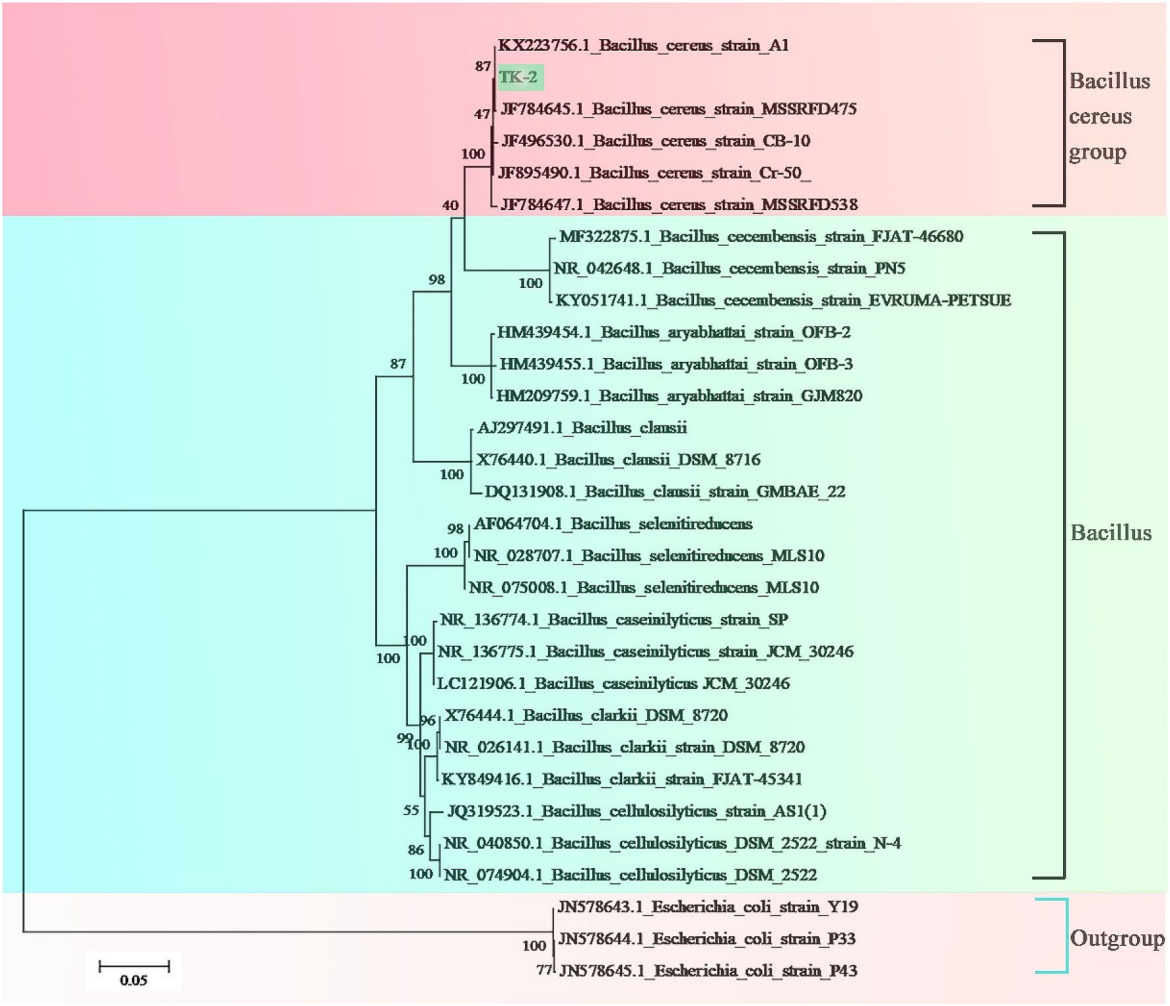
ML phylogenetic tree reconstruction containing the 16S rDNA genomes of 30 strains of bacteria. *Escherichia coli* was set as the outgroup.

A phylogenetic tree was reconstructed based on genomes of seven *Bacillus* groups, one *Bacillus cereus* group using the maximum likelihood (ML) method, with *Escherichia coli* as the outgroup. In the ML tree, all eight *Bacillus* species formed a robust monophyletic branch. Three *Bacillus* sp, *Bacillus cereus* A1, *Bacillus cereus* MSSRFD475 and *Bacillus* sp. TK-2—were clustered together in one terminal branch. Other seven *Bacillus* species are clustered together in one monophyletic group and nested in the branch of *Bacillus* groups (Fig. 5). The results confirmed that *Bacillus* sp. TK-2 is more closed to the *Bacillus* cereus group in the long-term evolution process. ANI is to determine the genetic relationship of two genomes at the nucleotide level by comparing the average base similarity between homologous fragments of two microbial genomes. It is characterized by a high degree of discrimination between closely related species^33^. ANI values of the genomes of *Bacillus* sp. TK-2 and other known *Bacillus cereus* strains in the NCBI database were calculated. It is found that *Bacillus cereus* TK-2 and *Bacillus cereus* strain A1 (GenBank: CP015727.1), isolated from the activated sludge of an anaerobic digestion reactor, had a high similarity of 98.26% (Table S1). *Bacillus cereus* strain A1 contained 5,667,342 bps, 34.9% GC content, 5699 protein-coding genes, 42 rRNAs and 105 tRNAs. It was used for the hydrolysis of starch and hydrogen production by fermentation of glucose at room temperature^34^. In this study, *Bacillus cereus* strain A1 was used as a reference genome, comparative genomics analysis containing common unique genes analysis, and mutation detection and annotation in these two strains were performed. The purpose of this work is to detect specific genes that may be involved in cold adaptation of *Bacillus* sp. TK-2 in a long-term cold environment.

**Figure 6:**
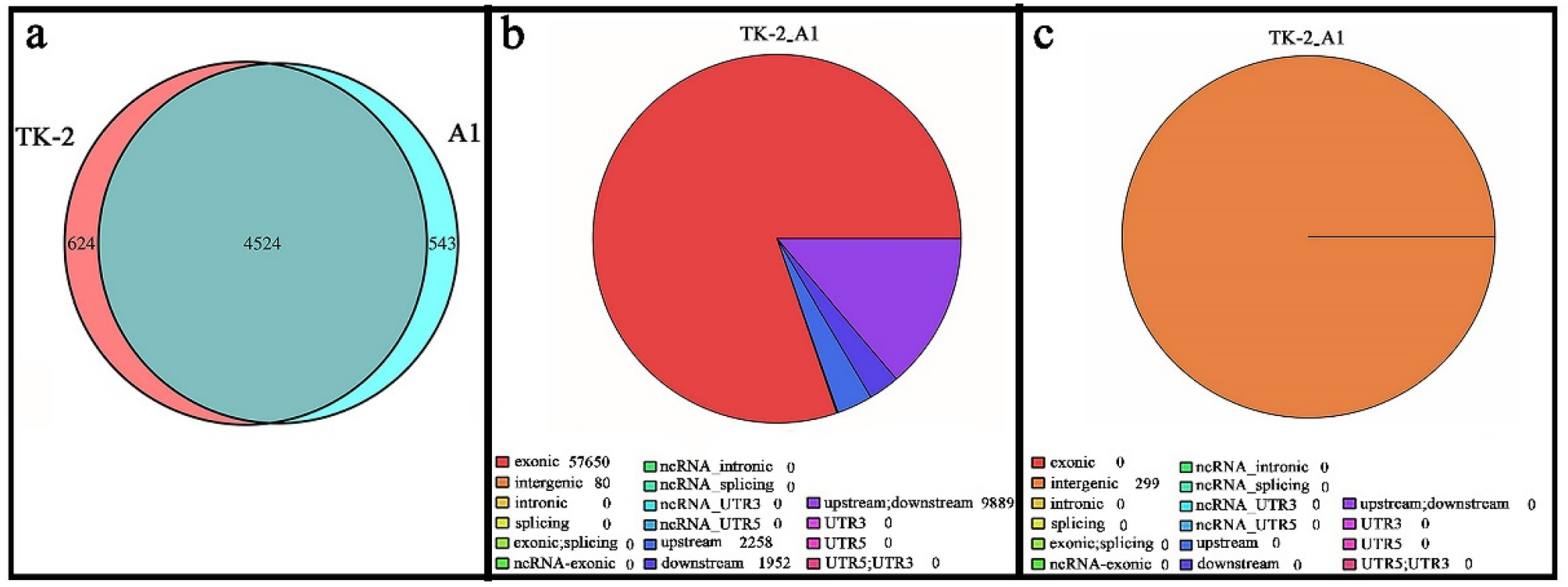
(**a**) Venn diagram of common and unique genes analysis. (**b**,**c**) Detection and annotation of SNPindel and structure.

Based on the statistical results, a Venn diagram was drawn (Fig. 6). It can be seen that pan genes have 5691 clusters, of which core genes have 4524 clusters, accounting for 79.5% of the total gene clusters, and *Bacillus* sp. TK-2 has 624 clusters for specific genes (Fig. 6a). The assembly result showed that there were two types of mutations, SNV/indel in these two strains by MUMmer, SNPindel detection and annotation, and the mutation sites mainly appeared in the exon region (57,650) and intergenic (80) (Fig. 6b). It was detected that large fragments of DNA indels (299) occurred in intergenic by structural detection and annotation (Fig. 6c). It can be seen that the two strains common genes, and there were mainly small fragments of gene rearrangements, deletions, and inversions in the unique genes of *Bacillus* sp. TK-2, which reflected the genomic commonality and uniqueness during the evolution of these two strains in different environments. Also, previously mentioned proteins that may participate in cold adaptation were found in 311 unique gene clusters of *Bacillus* sp. TK-2, mainly related to the synthesis of extracellular polysaccharides and unsaturation fatty acid, DNA replication, restriction/modification and repair, the metabolism of glutathione and glutathione spermidine, superoxide dismutase involved in antioxidant metabolism, and transcription regulators, the statistical results are shown in Table 3. Similar results were obtained Zhang et al., who provide evidence for the genetic basis of *Colwellia* sp. NB097-1 to survive in cold environments by its genome^35^. We will verify the function of these candidate genes related to cold adaptation and explore their role in the low-temperature evolution of *Bacillus* sp. TK-2 in future studies. Moreover, the performance of some genes with unknown functions at low temperatures is worthy of attention, which is of great significance for comprehensively revealing the evolutionary adaptability of *Bacillus* sp. TK-2.

**Table 3:** Statistics of genes related to cold adaptation among the unique genes of *Bacillus* sp. TK-2.

Substances or pathways	Genes ID
Synthesis of extracellular polysaccharide	WP_000599698.1
	WP_000916632.1
	WP_000140395.1
	WP_000250559.1
DNA replication, restriction/modification and repair	gnl|CDD|223435
Synthesis of unsaturated fatty acids	EC:1.14.19.2; 1.14.19.11 1.14.19.26
Glutathione spermidine metabolism	WP_098616269.1
Glutathione metabolism	ko00480
	WP_114160463.1
Superoxide dismutase	WP_114160463.1
Transcriptional regulator	WP_088338604.1

**Figure 7:**
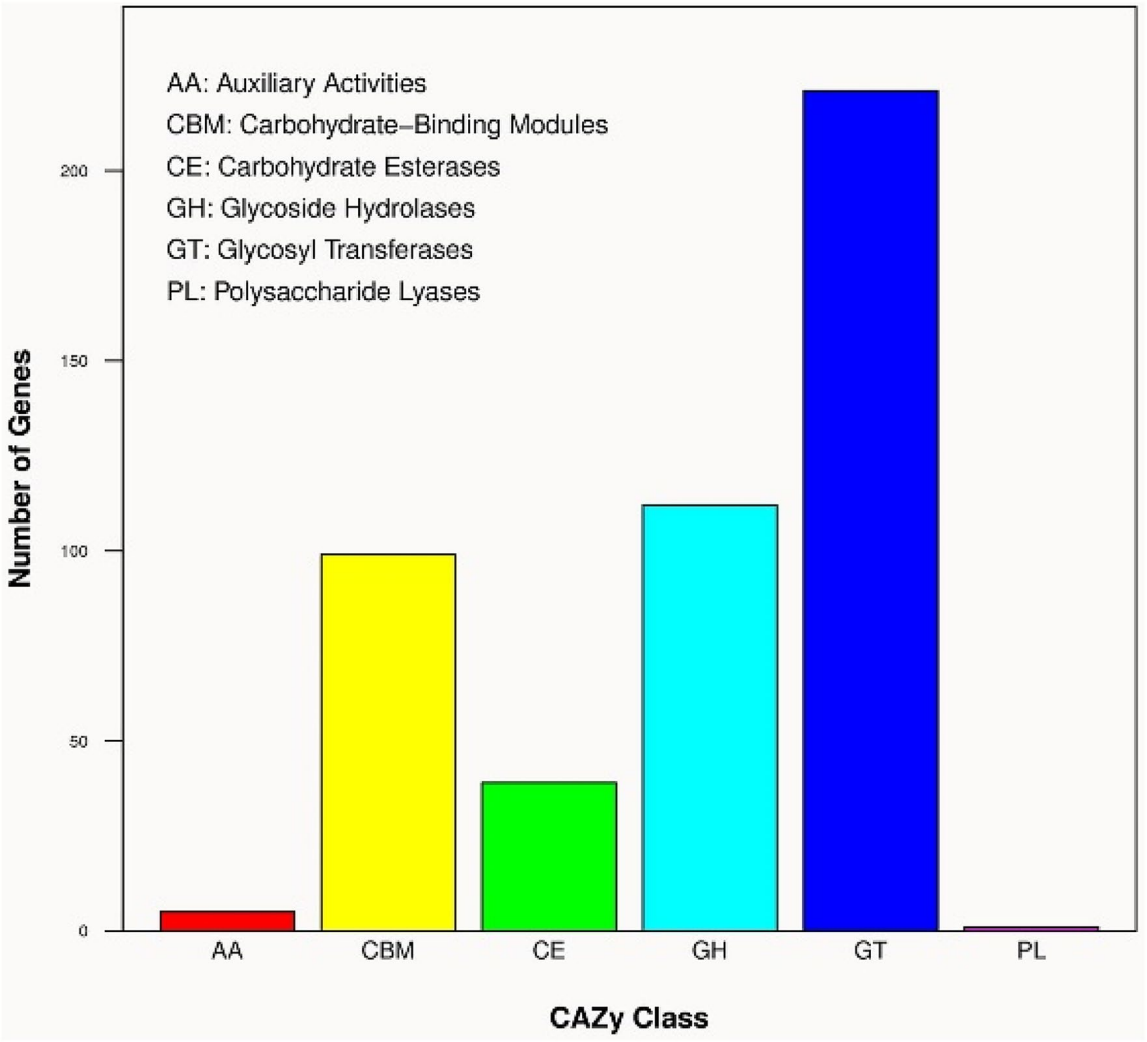
Gene distribution picture of CAZymes in *Bacillus* sp. TK-2.

The CAZy genes detected in this strain are classified into 6 categories by the CAZy (Carbohydrate-Active Enzymes) database annotation, including glycoside hydrolases (GH), glycosyl transferases (GT), polysaccharidelyases (PL), carbohydrate esterases (CE), carbohydrate-binding modules (CBM) and auxiliary activities (AA), which provide help for the analysis of the mechanism of carbohydrate utilization by this strain (Fig. 7). Among the 477 CAZyme genes annotated to function, 221 genes were found to belong to the GT family, including GT0, GT1, GT2, GT4, GT5, GT8, GT9, GT20, GT24, GT26, GT28, GT29, GT30, GT35, GT41, GT47, GT51, GT66, GT96.There are 99 genes in the CBM family, including CBM2, CBM3, CBM5, CBM9, CBM12, CBM13, CBM14, CBM20, CBM41, CBM47, CBM48, CBM50, CBM51, CBM54, CBM73. There are 5 genes in the AA family, including AA1, AA3, AA4, AA7, AA10. The PL12 family has only one gene. 39 genes are in the CE family, including CE0, CE1, CE4, CE9, CE11, CE14. 112 genes belong to the GH family, including GH0, GH1, GH2, GH3, GH4, GH5, GH8, GH9, GH12, GH13, GH16, GH18, GH19, GH23, GH24, GH25, GH28, GH32, GH33, GH39, GH47, GH53, GH65, GH72, GH73, GH84, GH92, GH93, GH99, GH109. Among these CAzyme genes, we focus on 8 genes encoding cellulase (GH3, GH5, GH8, GH9, GH12), 31 genes encoding hemicellulase (GH3, GH5, GH8, GH18, CBM9, CBM13). The existence of these genes has great potential for the degradation of biomass at low temperatures^5^.

## Methods

### Isolation of the strain TK-2 and extraction of genomic DNA

The strain TK-2 was isolated from Changbai Mountain's perennial low temperature soil, using salt solution (0.006 mM FeSO_4_·7H_2_O; 0.01 mM CaCO_3_·7H_2_O; 0.08 mM MgSO_4_·7H_2_O; 0.07 mM MnSO_4_·7H_2_O; 0.006 mM ZnSO_4_ 7H_2_O) and 1% saline buffer^36^. A single colony was inoculated into 100 mL of liquid LB medium (1 g tryptone, 1 g, 0.5 g yeast extract) at 160 rpm and 10° C for 3 days. A part of the bacterial solution was preserved at − 80 °C fridge by glycerol, and another part of the bacterial solution was used for genomic DNA extraction, strictly following the steps of the extraction kit (Shenggong Biological Co., Ltd., Shanghai, China). Genome quality and concentration were detected by agarose gel electrophoresis and NanoDrop. The medium and solution used in the experiment were sterilized at 121 °C for 20 min.

### Growth characteristics and identification of the strain TK-2

The growth characteristics of the strain TK-2 were evaluated by detecting its concentration (OD_600_) at different temperatures (10, 15, 20, 25, 37) °C. The species status of the strain TK-2 was determined by investigation of its morphology, physiological and biochemical characteristics and molecular biology. First, the color, shape, length, and surface morphology of the strain TK-2 were observed by eyes and microscope (fluorescence microanalysis system EVO FLC and scanning electron microscope JSM 6510LV). Secondly, some physiological and biochemical experiments (gram staining, fluorescent pigment, contact angle, pyocyanin, starch hydrolysis, indole production, gelatin liquefaction, hippuric acid, methyl red, VP determination, nitrate reduction, mannose alcohol production, etc.) were carried out^37^. After that, 16 s rDNA amplification and molecular identification were performed using the Sanger double-sided sequencing method. Fragments were amplified by using bacterial universal primers set 27F: AGAGTTTGATCCTGGCTCAG and 1492R: GGTTACCTTGTTACGACTT. The amplified fragments were subjected to DNA sequencing and compared with the known sequence homology in GenBank using nucleotide BLAST to determine the bacterial species. Finally, the strain TK-2 was assigned to a genus and species using the tests listed above (Genewiz Biotechnology Co., Ltd., Suzhou, China).

### Complete genome sequencing and annotation

The draft genome of the strain TK-2 is executed by Genewiz (Suzhou, China) using the Hiseq/Novaseq and Pacbio platforms. The library was sequenced using a PacBio RSII/Sequel SMRT instrument^38^. PacBio reads were assembled using HGAP4/Falcon of WGS-Assembler 8.2^39–44^. The genome was then corrected with Pilon 1.22^45^ using Quiver algorithm implemented in the Genomic Consensus package by default settings. The Prodigal 2.6.3^46^ gene-finding software was used for coding genes finding by default settings. Transfer RNAs (tRNAs) were detected in the genome using the program tRNAscan-SE^47^ with default settings. rRNA were identified by using RNAmmer^48^. The coding genes were annotated with the NR database using Diamond 0.8.15. Then the functions of genes were annotated with the GO database using Blast2go 2.5^48^, and the pathways were annotated by KEGG database using Blast 2.2.28+^49^. The proteins encoded by genes were classified on a phylogenetic classification by the database of COG using Hmmscan 3.1b2^50^. Enzymes related to carbohydrate degradation, synthesis and modification in the genome are annotated to describe catalytic structure and function information by CAZy using Diamond 0.8.15 (E-value 0.00001). The gene, ncRNA, GC content, and repeat sequence information were displayed on a circle diagram using Circos 0.69 software.

### 16s rDNA phylogenetic tree, ANI analysis, and comparative genomics analysis

Based on the 16s rDNA sequence of the strain TK-2 through blastn analysis results, A total of 30 complete 16s rDNA genome sequences were downloaded from the NCBI nucleotide resource database for phylogenetic analysis. *E. coil* was set as the outgroup, while MEGA6 was used to construct a phylogenetic tree using the ML method^51^. ANI analyses were performed to determine the relationship of *Bacillus* sp. TK-2 with other strains by their whole genome sequences. Based on the MUMmer alignment method, the average nucleotide identity (ANI) value between genomes was calculated^33^. Accession numbers of genomes of many *Bacillus* sp. strains could be discovered in GenBank.

Comparative genome analysis *Bacillus* sp. TK-2 and *Bacillus cereus* strain AI (GenBank: CP015727.1) with a high similarity of 98.26% was performed to discuss the unique cold survival characteristics of *Bacillus* sp. TK-2. The analysis content was mainly composed of three parts, including analysis of unique genes, SNPindel detection and annotation, and structure detection and annotation. Core genes (homologous genes present in all samples) and specific genes (differential genes for each sample) are likely to correspond to the commonality and characteristics of the samples, so they are used as the basis for the study of functional differences between samples. The sample cds sequence was clustered using cd-hit (Version 4.6). The clustering parameters and requirements are that the sequence similarity reaches 70%, the shortest sequence reaches 60% of the representative sequence length, and the alignment part reaches 60% of the sequence length. The reference genome sequence and gene bank files are used to construct the database. The target genome and reference genome are inputed into Anovar software for mutation site analysis. Based on the annotated gene information in the database, the mutation information was correlated with the gene information by the software to realize the annotation of mutation sites and the detection of amino acid changes caused by mutations. According to the result of the comparison between the assembly result and the reference genome Mauve, the insertion and deletion information of large DNA fragments was detected. The genome sequencing and bioinformatics analysis of *Bacillus* sp. TK-2 was performed by Genewiz Biotechnology Co., Ltd (Suzhou, China).

### Complete nucleotide sequence and strain accession numbers

The complete nucleotide sequence of *Bacillus* sp. TK-2 was deposited in GenBank database under the accession number of CP067375. The strain can be obtained from Key Laboratory of Straw Biology and Utilization, The Ministry of Education, China, Changchun, 130118.

## Supplementary Information

Supplementary Table.

Supplementary Figures.

## Data Availability

All data generated or analysed during this study are included in this published article.
