# Supplementary material for: Complete genome sequencing of Bacillus sp. TK-2, analysis of its cold evolution adaptability
Source: Sci Rep. 2021 Mar 1;11:4836. doi: 10.1038/s41598-021-84286-7 (PMC7921382; doi:10.1038/s41598-021-84286-7)
Supplement: Supplementary file 1 — Supplementary Table. [file 41598_2021_84286_MOESM1_ESM.docx]

**Table S1. ANI values (%) generated by comparing the genomes of *Bacillus* sp. TK-2 and 14 *Bacillus cereus* strains.**

|  | Total bases | ANI value | Accession number |
| --- | --- | --- | --- |
| *Bacillus cereus* strain A1 | 5352307 bp | 98.26 | CP015727.1 |
| *Bacillus cereus* NC7401 | 5221581 bp | 90.99 | AP007209.1 |
| *Bacillus cereus* Rock1-15 | 5766278 bp | 97.06 | CM000729.1 |
| *Bacillus cereus* AH1271 | 5656704 bp | 90.90 | CM000739.1 |
| *Bacillus cereus* AH676 | 5594747 bp | 96.96 | CM000738.1 |
| *Bacillus cereus* 95/8201 | 5584055 bp | 90.77 | CM000727.1 |
| *Bacillus cereus* BDRD-Cer4 | 5397450 bp | 96.99 | CM000726.1 |
| *Bacillus cereus* BGSC 6E1 | 5730178 bp | 91.08 | CM000716.1 |
| [*Bacillus cereus* AH1273](https://www.ncbi.nlm.nih.gov/nuccore/CM000741.1) | 5790501 bp | 89.50 | CM000741.1 |
| *Bacillus cereus* Rock3-42 | 5202904 bp | 90.78 | CM000732.1 |
| *Bacillus cereus* BDRD-ST24 | 5436173 bp | 96.90 | CM000723.1 |
| *Bacillus cereus* ATCC 4342 | 5234797 bp | 91.28 | CM000721.1 |
| *Bacillus cereus* R309803 | 5586253 bp | 90.55 | CM000720.1 |
| *Bacillus cereus* E33L | 5300915 bp | 90.78 | CP000001.1 |
