# Supplementary material for: Complete genome sequencing of Bacillus sp. TK-2, analysis of its cold evolution adaptability
Source: Sci Rep. 2021 Mar 1;11:4836. doi: 10.1038/s41598-021-84286-7 (PMC7921382; doi:10.1038/s41598-021-84286-7)
Supplement: Supplementary file 2 — Supplementary Figures. [file 41598_2021_84286_MOESM2_ESM.pdf]

Complete genome sequencing of *Bacillus* sp. TK-2, analysis of its cold evolution adaptability

Lijun Shen, [a,b] Xueli Zang, [c] Ke Sun, [d] Huan Chen, [a,b] Xinying Che, [a] Yang Sun, [a,b] Gang Wang, [a,b]

Sitong Zhang, [a,b,*] and Guang Chen [a,b,*]

[a]*College of Life Sciences, Jilin Agricultural University, Changchun, China*

[b]*Key Laboratory of Straw Biology and Utilization, The Ministry of Education, Changchun, China*

[c]*School of Medicine and Food, Changchun Medical College, Changchun, China*

[d]*COFCO Biochemical Energy (Gongzhuling) Co., Ltd., Changchun, China*

*Corresponding author, e-mail: 18943132269@163.com (ST. Zhang),

chg61@163.com (G. Chen)

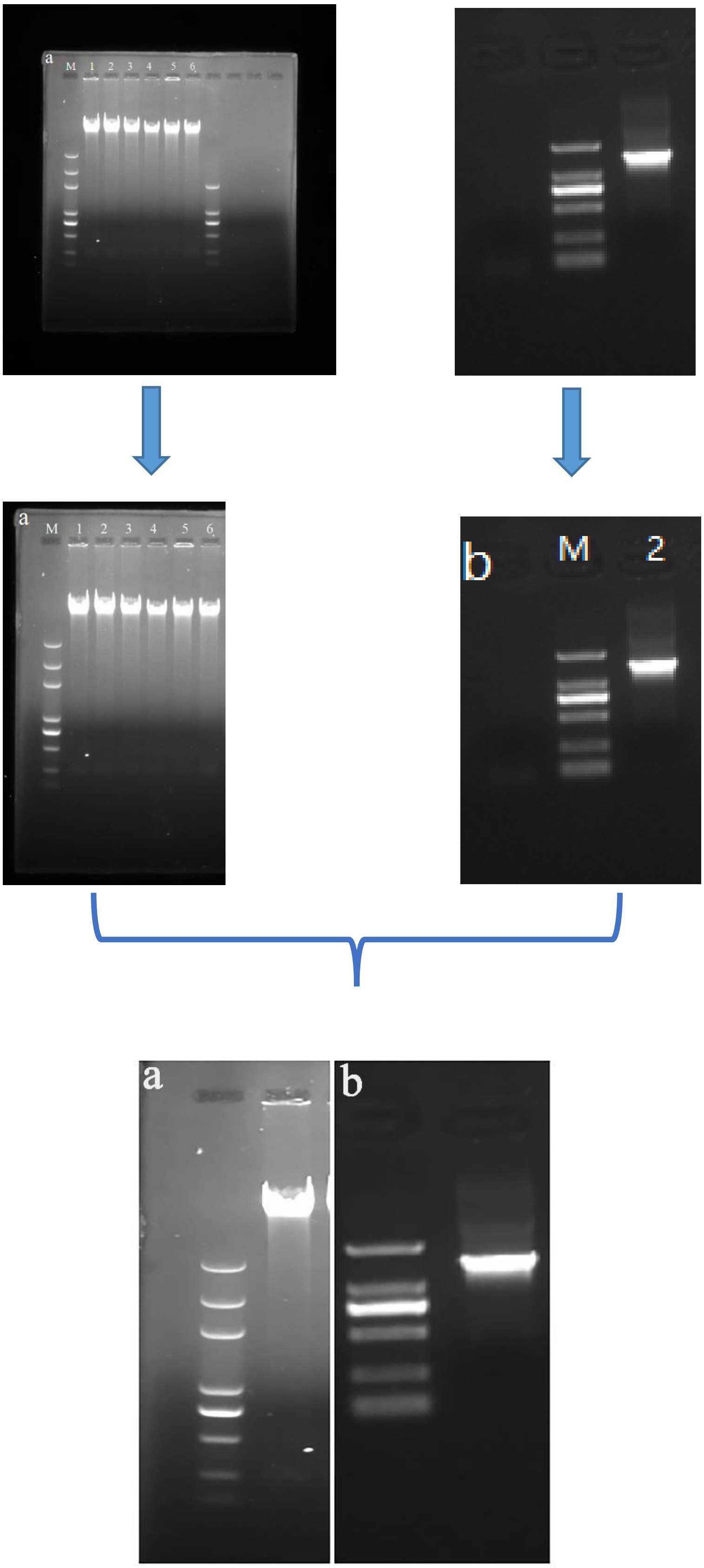
a
M 1 2 3 4 5 6
a
M 1 2 3 4 5 6
b
M 2
a
b

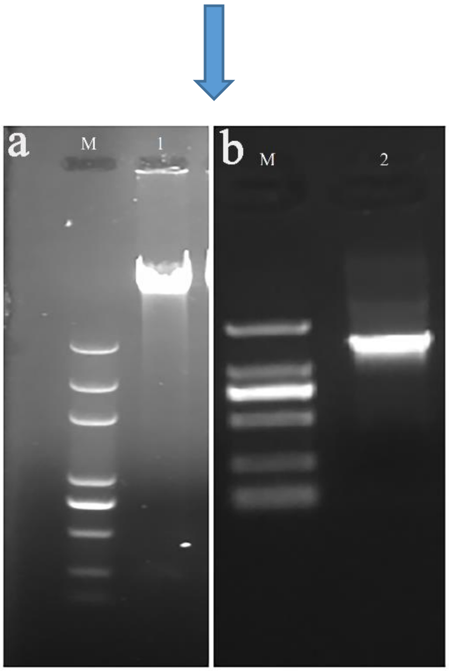

Figure S1. Agarose gel electrophoresis. (a) DNA extracted from *Bacillus* sp. TK-2. M(Marker): (DL15000：15000、8000、5000、3000、2000、1500、1000、750bp), 1: TK-2. (b) PCR product of 16S rDNA of *Bacillus* sp. TK-2. M(Marker): (DL2000：2000、1000、750、500、250、100bp), 2: TK-2.

```
   1 tgcagtcgag cgaatggatt aagagcttgc tcttatgaag ttagcggcgg acgggtgagt
  61 aacacgtggg taacctgccc ataagactgg gataactccg ggaaaccggg gctaataccg
 121 gataacattt tgaactgcat ggttcgaaat tgaaaggcgg cttcggctgt cacttatgga
 181 tggacccgcg tcgcattagc tagttggtga ggtaacggct caccaaggca acgatgcgta
 241 gccgacctga gagggtgatc ggccacactg ggactgagac acggcccaga ctcctacggg
 301 aggcagcagt agggaatctt ccgcaatgga cgaaagtctg acggagcaac gccgcgtgag
 361 tgatgaaggc tttcgggtcg taaaactctg ttgttaggga agaacaagtg ctagttgaat
 421 aagctggcac cttgacggta cctaaccaga aagccacggc taactacgtg ccagcagccg
 481 cggtaatacg taggtggcaa gcgttatccg gaattattgg gcgtaaagcg cgcgcaggtg
 541 gtttcttaag tctgatgtga aagcccacgg ctcaaccgtg gagggtcatt ggaaactggg
 601 agacttgagt gcagaagagg aaagtggaat tccatgtgta gcggtgaaat gcgtagagat
 661 atggaggaac accagtggcg aaggcgactt tctggtctgt aactgacact gaggcgcgaa
 721 agcgtgggga gcaaacagga ttagataccc tggtagtcca cgccgtaaac gatgagtgct
 781 aagtgttaga gggtttccgc cctttagtgc tgaagttaac gcattaagca ctccgcctgg
 841 ggagtacggc cgcaaggctg aaactcaaag gaattgacgg gggcccgcac aagcggtgga
 901 gcatgtggtt taattcgaag caacgcgaag aaccttacca ggtcttgaca tcctctgaaa
 961 accctagaga tagggcttct ccttcgggag cagagtgaca ggtggtgcat ggttgtcgtc
1021 agctcgtgtc gtgagatgtt gggttaagtc ccgcaacgag cgcaaccctt gatcttagtt
1081 gccatcatta agttgggcac tctaaggtga ctgccggtga caaaccggag gaaggtgggg
1141 atgacgtcaa atcatcatgc cccttatgac ctgggctaca cacgtgctac aatggacggt
1201 acaaagagct gcaagaccgc gaggtggagc taatctcata aaaccgttct cagttcggat
1261 tgtaggctgc aactcgccta catgaagctg gaatcgctag taatcgcgga tcagcatgcc
1321 gcggtgaata cgttcccggg ccttgtacac accgcccgtc acaccacgag agtttgtaac
1381 acccgaagtc ggtggggtaa cctttt
```

Figure S2. Nucleotide sequence of 16S rDNA from *Bacillus* sp. TK-2.
